# A Cluster-Based Energy-Efficient Secure Optimal Path-Routing Protocol for Wireless Body-Area Sensor Networks

**DOI:** 10.3390/s23146274

**Published:** 2023-07-10

**Authors:** Ruby Dass, Manikandan Narayanan, Gayathri Ananthakrishnan, Tamilarasi Kathirvel Murugan, Musiri Kailasanathan Nallakaruppan, Siva Rama Krishnan Somayaji, Kannan Arputharaj, Surbhi Bhatia Khan, Ahlam Almusharraf

**Affiliations:** 1 School of Computer Science and Engineering, Vellore Institute of Technology, Vellore 632014, India; ruby.d@vit.ac.in (R.D.); mkyadhav@yahoo.com (M.N.); kannan.a@vit.ac.in (K.A.); 2 School of Information Technology and Engineering, Vellore Institute of Technology, Vellore 632014, India; gayathri.a@vit.ac.in (G.A.); nallakaruppan.mk@vit.ac.in (M.K.N.); siva.s@vit.ac.in (S.R.K.S.); 3 School of Computer Science and Engineering, Vellore Institute of Technology, Chennai 600127, India; 4 Department of Data Science, School of Science, Engineering and Environment, University of Salford, Manchester M5 4WT, UK; surbhibhatia1988@yahoo.com; 5 Department of Electrical and Computer Engineering, Lebanese American University, Byblos 13-5053, Lebanon; 6 Department of Business Administration, College of Business and Administration, Princess Nourah bint Abdulrahman University, P.O. Box 84428, Riyadh 11671, Saudi Arabia

**Keywords:** wireless body-area network, routing, energy efficiency, black-hole attack

## Abstract

Recently, research into Wireless Body-Area Sensor Networks (WBASN) or Wireless Body-Area Networks (WBAN) has gained much importance in medical applications, and now plays a significant role in patient monitoring. Among the various operations, routing is still recognized as a resource-intensive activity. As a result, designing an energy-efficient routing system for WBAN is critical. The existing routing algorithms focus more on energy efficiency than security. However, security attacks will lead to more energy consumption, which will reduce overall network performance. To handle the issues of reliability, energy efficiency, and security in WBAN, a new cluster-based secure routing protocol called the Secure Optimal Path-Routing (SOPR) protocol has been proposed in this paper. This proposed algorithm provides security by identifying and avoiding black-hole attacks on one side, and by sending data packets in encrypted form on the other side to strengthen communication security in WBANs. The main advantages of implementing the proposed protocol include improved overall network performance by increasing the packet-delivery ratio and reducing attack-detection overheads, detection time, energy consumption, and delay.

## 1. Introduction

**Figure 1:**
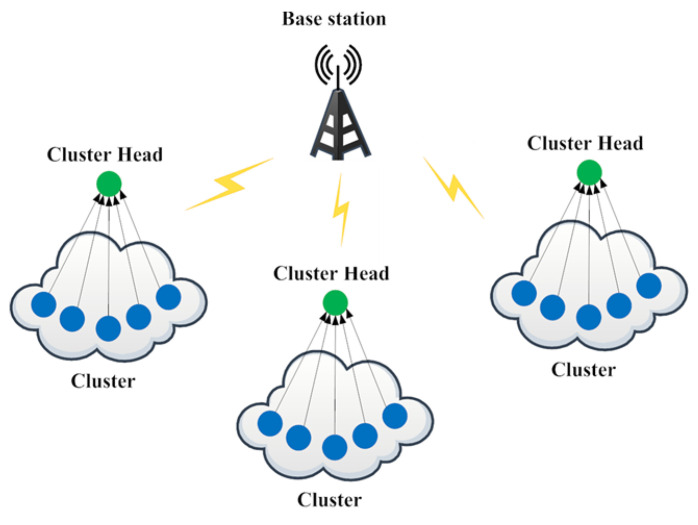
WSN without and with clustering.

A Wireless Sensor Network (WSN) is a self-adaptive network consisting of sensor nodes that typically have limited processing, memory, and battery power. These sensor nodes are interconnected via a wireless medium such as Bluetooth, Zigbee, and Wi-Fi. A WSN consists of three types of nodes: a sink node, a routing node, and a sensor node [1]. From a cluster node, the sink node collects data and transfers it to the server node via intermediary routers. The routing nodes can be individual routing devices or one of the sensor nodes themselves. To decrease the number of packets sent over the network, a group of nodes identifies the cluster head (CH) and aggregates the data, other than those from the sink nodes. Figure 1 shows the data transmission between the source node and base station using clustering [2,3]. Cluster heads are designated based on node reliability, base station distance, and residual energy. They must have the highest residual energy and be within a one-hop distance of the base station [4,5].

A subsection of Wireless Sensor Networks known as Wireless Body-Area Networks (WBAN) involves the continuous monitoring of health constraints in everyday life. The WBAN system consists of small sensors that are fixed to various parts of the human body to monitor functional parameters. In addition to recording data, each sensor can transfer them to the sink and the base station. Security of transmission is also a major constraint of WBAN because wireless media is open-access. Black-hole attacks are one of the most common attacks in WBAN, which involve the capture of these nodes by malicious intruders and the re-programming of them to drop packets or produce false packets to break the network. Therefore, it is obligatory to propose a cluster-based and energy-efficient secure routing protocol to enhance the reliability of communication in WBAN.

To tackle the threats posed by malicious nodes through black-hole attacks, a new cluster-based energy-efficient and secure routing protocol called Secure Optimal Path-Routing (SOPR) is proposed in this paper to provide reliable and secure routing in WBAN. This protocol employs two algorithms, namely the Balanced Energy-Efficient and Reliable (BEER) algorithm and the One-Time Password and Shift Operations-based encryption and decryption algorithm, which uses a nonce (random number used to safeguard private communications by averting replay attacks), as well as an energy-modeling approach, which has also been suggested in this work to improve security and reduce energy consumption, leading to an increase in network lifespan. The encryption algorithm proposed in this work also performs node authentication to recognize malicious nodes and prevent them from communicating or routing in the network. The nodes in the network are clustered with a designated cluster head (CH) chosen for each cluster with high energy and without a history of malicious activities, including the creation of black holes and the regular dropping of packets.

The route-discovery process is initiated by the source sensor node, and its cluster head leads the source node. The reliable, secured, and shortest path from the source to the destination is found by flooding the packets through the cluster-head nodes. This continues until the first route-request packet reaches the destination. The destination directs the route-reply packet to follow the same path as the first route-request packet. In this study, the energy-efficient and optimal path was identified through the application of SOPR, BEER, and OTP-based encryption and decryption algorithms. These proposed strategies were tested using the NS2 simulator and compared to the existing energy-efficient and secure routing protocols. Simulations revealed that the proposed WBAN protocol improved the performance of the network, including throughput, packet-delivery ratio, and security when compared to traditional protocols. Furthermore, the proposed protocol decreased the energy consumption, delay, and time required to detect malicious nodes.

### 1.1. Contributions of the Work

The main contributions of this work are listed below:

- A Secured Optimal Path-Routing (SOPR) protocol for improving the performance of wireless body-area networks is proposed.
- This protocol offers improved packet-delivery ratio, enhanced security, and reduced attack-detection overhead, detection time, energy consumption, and delay compared to existing protocols.
- In addition, this protocol can be applied to different wireless body-area networks, such as those used in healthcare applications or environmental monitoring, to ensure secure and energy-efficient routing.

### 1.2. Organization of the Work

The remainder of this paper is structured as follows: Section 2 outlines the relevant works on clustering, cluster-based routing, energy efficiency, and encryption/decryption-based and attack-detection-based secure routing algorithms. Section 3 explains the proposed systems by highlighting the newly proposed algorithms. In Section 4, the proposed system’s overall performance is evaluated by comparing it with the existing systems based on results. Section 5 discusses the results obtained with respect to different parameters. Section 6 provides conclusions to this work, and additionally recommends some potential future works.

## 2. Materials and Methods

In [6], the selection of the cluster head is based on traffic priority data and energy levels, followed by an optimal route for data transmission. The authors in Ref. [7] proposed a novel algorithm called Power-Efficient Cluster-based Routing (PECR), which uses K-Means clustering, optimal route choice, communication based on energy use, cluster head and primary cluster head alternation for energy efficiency and increased network lifespan. To evaluate the performance of EEDLABA, an energy-consumption model and path loss are proposed, with nine sensor nodes deployed on a human body [8]. There are many research studies on routing protocols for WSN with energy efficiency, security, and cluster-based routing [9,10,11,12,13,14]. In this direction, the authors in Ref. [15] proposed a Feedback Routing to Multiple Sinks (FROMS), with a multicast routing protocol based on reinforcement learning and modified transmission back-offs and acknowledgments. With the proposal, the authors have shown that machine-learning algorithms can be successfully applied to devices with limited constraints. Hu et al. [16], overcame the problems of propagation delay and power consumption using a machine-learning approach for routing wireless sensor nodes underwater. This method proposed a balanced routing protocol called the Q-learning-based Energy-Efficient and Lifetime-Aware Routing Protocol (QELAR). It distributes routing across all sensor nodes. Hierarchical routing protocols were used to exploit the energy of the sensor nodes. Based on the above concept, an energy-efficient shortest-path Q-Routing algorithm was proposed in [17]. This algorithm is based on reinforcement learning, which extends the network lifetime.

Dynamic topology, one of the characteristics of WSN networks, has complicated the routing mechanism. Another challenge is to minimize resource consumption during routing. A Support Vector Machine-based clustering method was proposed in [18]. To reduce energy consumption, the proposed method allocates the nodes to the closest cluster head. A Naive Bayesian-based classification method was suggested in [19] to predict traffic load and energy in the selected path. Estimating the link cost of nodes is one of the key tasks in routing. To calculate the link cost, the authors in [20] proposed an approach based on machine learning. They evaluated the performance of the algorithms using various machine-learning techniques. To enhance network lifetime, a Hybrid Clustering Routing Protocol–Hole Detection (HCRP-HD) was proposed by Masoud et al. [21] by detecting holes and edge nodes. The sink node is responsible for hole and edge detection, reducing the energy consumption of the sensor node. The network is transformed into several rings to reduce energy consumption due to direct transmission.

In [22], the author proposed a machine-learning-based User-Specific Optimal Capacity Shortest-Path (US-OCSP) routing to find the shortest path. Here, they have considered the available capacity in the nodes and distance to determine the optimal path between the source and destination. The congested nodes are avoided using the Q-learning algorithm to improve the throughput and bitrate of the network. Most of the routing algorithms require high bandwidth to manage the routing table entries or avoid suffering from high delay. To overcome this, the route discovery is done on demand, and the update to the route is done proactively. A novel Q-learning-based algorithm was proposed by the authors in [23] by incorporating QoS in the existing reinforcement-learning-based algorithms. The packets in different traffic classes will be routed via different routes; therefore, this method provides high priority for important packets. An Energy-Centric Route-Planning (ECRP) method was introduced in [24] to balance the sensor node lifetime. It also ensures security along with the routing. To maintain a balanced communication channel, the energy requirements of individual and cooperative nodes were taken into consideration. It is very difficult to identify non-trusted nodes in wireless networks dynamically. A reinforcement learning-based scheme combined with Blockchain technology to enhance efficiency and security during routing was proposed in [25]. The routing information is stored in the Blockchain; therefore, this information is immutable. Reinforcement learning is used to select more trusted and efficient links dynamically.

A novel load-balanced routing protocol based on machine learning was proposed by the authors of [26]. To make intelligent routing decisions, the adjacency matrix of the network topology is first reduced using Principal Component Analysis, and the status of the network queue is predicted using neural networks for intelligent routing decisions. The load-balancing routing algorithm is implemented considering the Queue Use. The shortest path and link stability should be considered to decrease the end-to-end delay. To predict the behavior pattern of the network nodes, a method based on Q-learning was proposed in [27]. The authors in [28] suggested a genetic algorithm-based routing protocol for efficient routing. The unhealthy nodes were identified, and energy use and computational time were reduced. To improve network lifetime, a clustering-based routing protocol was proposed by the authors in [29]. The entire network is segmented into clusters, and each cluster has a designated cluster head through which all the network nodes transport the data. The authors in [30] presented a Multi-Objective Multi-Hop Routing (MOMHR) protocol to improve network lifetime by ensuring optimal routing. This protocol works in two phases. In the first phase, K-Means clustering is used to cluster the entire network. To identify the best cluster head in each cluster, an artificial bee-colony optimization algorithm is used in the second phase. Then, the multi-objective function is used to find a low-cost route from a node to a base station.

This approach has been further enhanced by the authors in [31] by introducing a trust-based routing for secured routing. An optimization algorithm called Chicken–Dragonfly (CHicDra) was proposed to find the optimal cluster head. The finalization of the trusted nodes is undertaken with the inclusion of parameters such as integrity factors, consistency factors, forwarding rate factors, and availability factors. The authors in [32] proposed a Residual Energy-based Cluster-Head selection along with the LEACH algorithm (RCH-LEACH) by taking into account parameters such as threshold energy, residual node energy, and the optimal number of clusters. Relative inter-cluster and intra-cluster costs are presented in [33], and they proposed a multi-hop unequal clustering scheme based on fuzzy logic that reduces the energy requirement of cluster members. A node may decide on its own, based on the probability mechanism. They also introduced a self-adaptive rotation mechanism that reduces re-clustering. Enhanced Hybrid Multipath Routing (EHMR) was proposed by the authors in [34], based on the hierarchical clustering technique. To route a packet, the next hop is identified by minimum hop count and maximum residual energy. Load-balancing and fast failure recovery mechanisms were also proposed.

Due to node replication, node attacks are inevitable in WBAN. A new type of walk called Solo Stage Random Walk Memory with a Distributed Network was proposed by Aalsalem et al. [35] to address this issue; it ensures a node’s security while maintaining a reasonable memory and communication overhead. Data communication is not secure in both directions, because nodes are vulnerable to various attacks. Even though data communication is guaranteed, higher network densities are said to impair attack detection. Several types of sinkhole nodes have been identified to address this issue by the authors in [36] based on numerous disjoint clusters. Several solutions are provided to secure WBAN from black-hole attacks. Such attacks on the sensor network are protected using AI techniques such as the hierarchically efficient Intrusion Detection System (IDS) proposed by the authors of [37,38,39,40]. This approach relies on the interchange of control packets between the base station and the sensor node. However, its security remains unresolved. Good studies have been completed on Blockchain’s support of the use of this type of security. When processing a huge number of transactions, validating multiple signatures incurs unnecessary overheads on the nodes of the Blockchain [41]. Open communication between doctors and patients is another benefit of the direct mode. However, this raises concerns about privacy and security due to vulnerabilities such as man-in-the-middle, sniffing, and pursuit attacks [42,43].

Despite all this work in the literature, existing systems have many limitations. First, most of the existing protocols send the data in plain text form without encryption. Second, the existing systems do not check for the presence of the malicious nodes that perform black-hole attacks. Third, energy modeling is not considered in all the studies. Finally, clustering and load-balancing issues are not considered in the existing works. Therefore, this paper proposes a cluster-based energy-efficient secure routing protocol that encrypts the packets on the sender side and decrypts them on the receiver side to improve the security, energy efficiency, and reliability of communication in WBAN.

## 3. Proposed Methods

**Figure 2:**
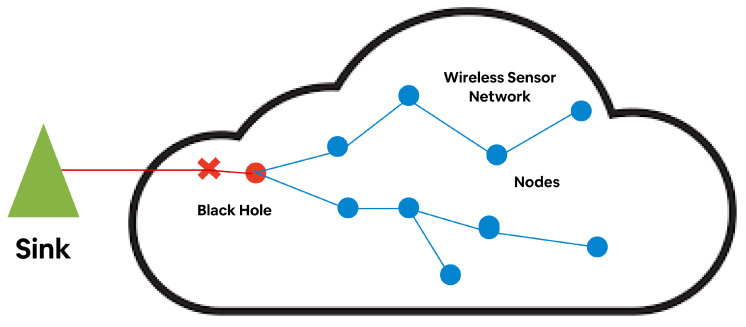
Black-hole attack.

The research methodology proposed in this work implements black-hole attack detection along with a design for protection using routing by the Secured Optimal Path-Routing (SOPR) protocol in WBANs. Figure 2 depicts the protection technique and also identifies the minimal path between the source and sink. Here, we presume the source and sink are not malicious. The proposed technique gives suggestions for identifying malicious routes using the routing protocol SOPR, interrelating with the Balanced Energy-Efficient and Reliable (BEER) algorithm, which offers enhanced energy. The SOPR protocol is incorporated with encryption along with routing.

Protection from a black-hole attack is performed in three phases. Initially, the network is noted by employing the sensor nodes. In stage 2, the functions of the proposed algorithm are operated through the evaluation of a minimal path between the source and sink using the proposed SOPR algorithm. The forwarding rate or trust value of every node through every route is used to detect the black-hole attack on the network. Therefore, every route has been taken into account for the SOPR state in this proposed design. The final stage in SOPR, along with encryption using a one-time pad, is incorporated for the evaluation of the maximum possibility for security in routing. Because of this mechanism, a route based on trust has been acquired by the hash function generation of this encryption technique. The one-time pad is used for the encryption and decryption process when selecting a secured path for transmitting the data.

**Figure 3:**
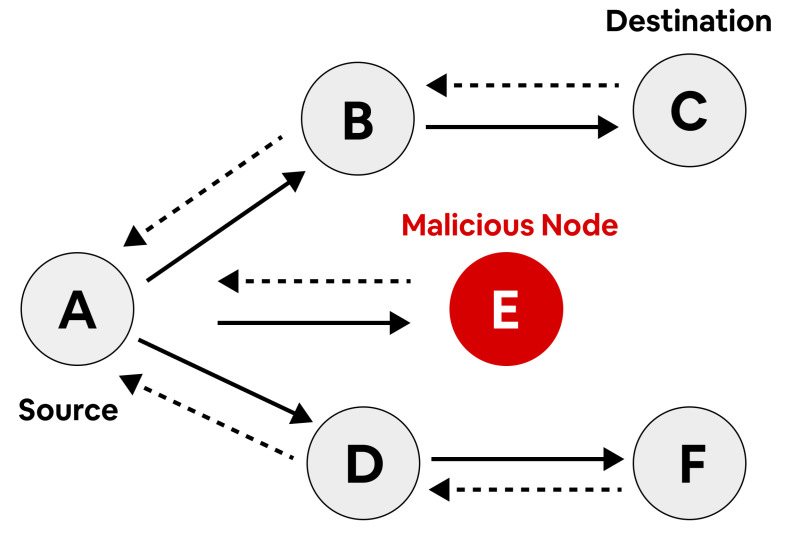
Schematic illustration of a black-hole attack using REQR and REPR packets. A–D, F denotes nodes, Solid arrows denotes REQR and dotted arrows denotes REPR.

All the data have been captured, including all the malicious nodes in the WBAN, by the black-hole node. Therefore, the fake responses have been forwarded through the path, which either blocks or drops the packet rather than forwarding true information along the active path. This scenario is presented in Figure 3. Due to this constraint, the source cannot communicate through the sink node. Although the source sensor node requires communication with the sink node, every node in the network receives the request message. The malicious node (E) replies fake REPR to the source node with a lower hop count number that could reach the sink node. Thus, a black-hole attack is executed within the network.

The proposed technique for protecting the network from attack is discussed below.

### 3.1. Formation of Key Encryption for Plain Text

In every round of transmission, the novel key for encryption is generated for every sensor node. Here, we use a one-time pad for the encryption and decryption process. In the sensor network, three sensor node types are employed, namely stage 0, stage 1, and stage 2 sensor nodes. The nodes of stage 1 and stage 2 are employed without manual support. The node distribution for stage 1 and stage 2 remains constant. The total number of sensor nodes in stage 1 is 10–20% and for stage 2 is 20–30%. The initial key globally represented as K0 for stage 0, stage 1, and stage 2 has been preloaded. Moreover, a separate secret key has been allotted through the destination node that is held by every node. Individual identity (ID) has been given for every node as well as the operation of pseudo-random value f, which is preloaded. The destination node is presumed to be secure as well as trusted. Although a minimal bound of time interval Tmin has been assumed, this remains essential by the opposite phase to negotiate with the node. The cluster heads initiate localizing self-organization through the transmission of a hello message <IDC|NC|MAC (K0, IDG|NG)> by the stage 1 node, where IDC is the group leader’s ID and NC denotes the nonce. The initial verified hello message by the Medium Access Control (MAC) is accepted through every node (say, node X). The transmitter as its parent locates the destination as X. <IDX|IDC|NC|MAC (K0, IDX|IDC|NC)> is sent to the destination as the response message. The <IDX|IDC|NX|MAC(K0, IDX|IDC|NX)> message is transmitted as the hello message, which is updated. By the MAC layer, the message which is given as a response has been accepted by the cluster head, and node X is allotted as their child node. These functions are carried out recurrently. The termination of the hello message transmission is made when the hello message is received by the sensor node. The transmission is halted when the neighbor cluster’s head broadcasts the message sooner. Every cluster head has its identity as ID, which is reported to every other cluster head. This ends the process of localizing self-organization. When this process ends, the cluster head, which acts as a base, has coordinated with the nodes through the design of the tree. Every cluster head comprises the collected data, namely (a) the ID of the neighbor’s one-hop, and (b) the ID of the neighbor’s cluster head. In other sensor nodes, the data accumulated comprises (a) the ID of the parent node, (b) the ID of the child node, and (c) the ID of the cluster head. At first, the secret key of the cluster head f K0 (ID) is derived on its own. The cluster head of the neighbor will derive f K0 (ID), the private key, where ID is the ID of the neighboring cluster head. After secret key derivation, the generation of a shared key for the inter-cluster by the cluster head of the neighbor takes place. We assume the cluster heads to be X0 and Y0. The generation of the shared key KXY is given here. If X0 ID ≥ Y0 ID, the pair-wise shared key KXY = fK IDX has been generated by node X0. Node Y0 computes KXY as KXY = f K IDX independently. Finally, the time has elapsed, and every node can reject the destination node (K0, f) along with the secret key of the nodes. The message sent by the cluster head is ID|IDR|MAC (K0, ID|IDR) for their single-hop nearest clusters, and a random identity is given as IDR. The message authentication of the MAC is validated through the nearest clusters. Intra-cluster key-sharing has taken place among the nearest cluster key f K IDR. Therefore, the intra-cluster key-sharing is only for the nearest cluster head.

### 3.2. OTP Algorithm

The OTP algorithm parameters are described in Table 1.

**Figure 4:**
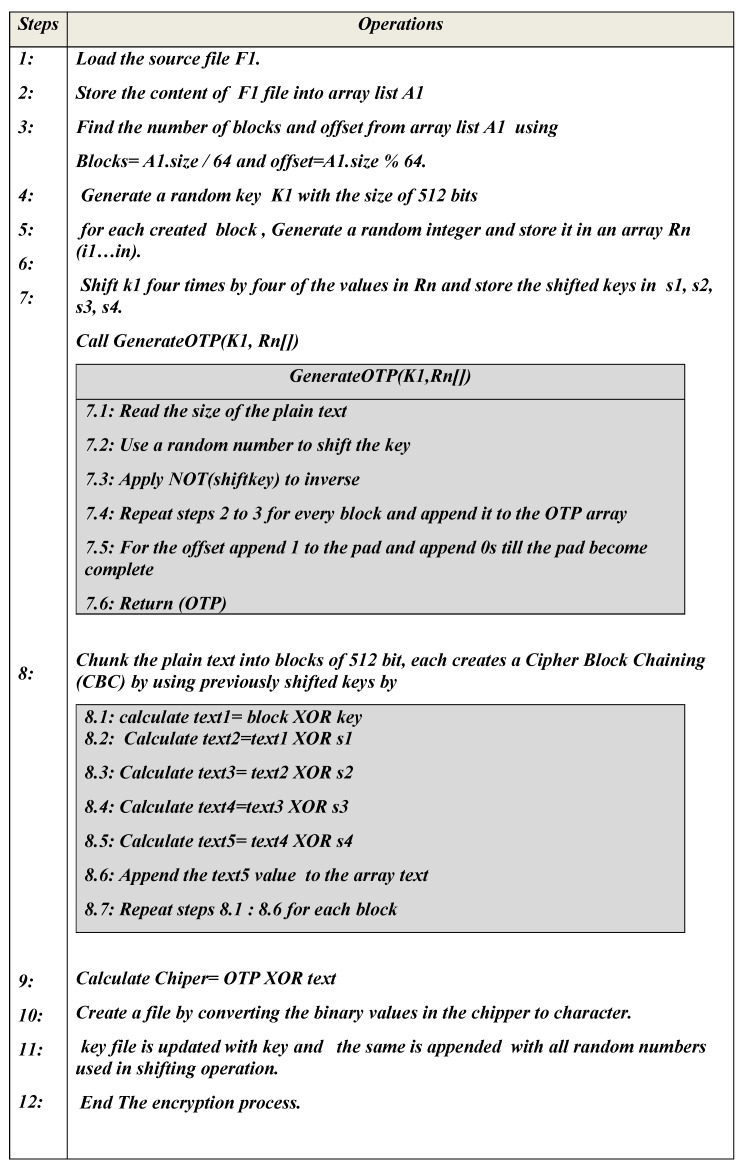
Encryption process of the OTP algorithm.

The overall encryption algorithm is described in Figure 4.

**Figure 5:**
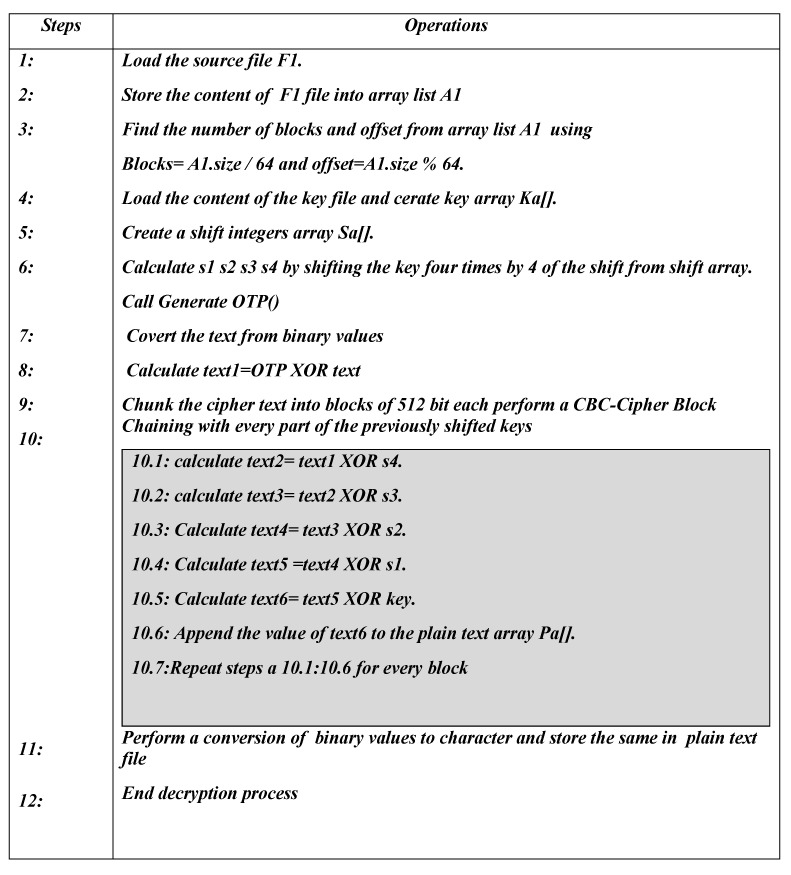
Decryption process of the OTP algorithm.

The overall decryption algorithm is described in Figure 5.

### 3.3. Secure Optimal Path-Routing Protocol

With the process of localizing the self-organization as well as with the function of key generation, the network has been classified into clusters. Every cluster has a cluster head. The key-sharing for the intra-cluster between the cluster heads of the nearest clusters and the key-sharing for the inter-cluster between the subsequent cluster head of the nearest clusters are generated. In the proposed SOPR, the transmitter node creates the operation of route discovery by transmitting the request for the route (REQR) to the receiver node. When the receiver node and their central cluster head possess the probable route, they will accept the REQR, create the reply for the route (REPR), and send it back to the transmitter node. Every REPR comprises the acknowledgment code for the message, which is known to be a MAC that has been evaluated through the key-sharing of the inter-cluster. Every REPR packet has two stages of verification, which are simulated as follows: initially, the cluster head for the next cluster is created. The REPR packet is validated by sending a pending authentication message from the cluster head to the next hop en route to the receiver node. The MAC, which has been verified through the intra-cluster key exchange, makes up the authentication message. The authentication message accepted by the next hop sends the authentication output to the transmitter. When this process is accomplished, the node forwards the REPR packet of those data. Otherwise, when the authentication is not accomplished or the node cannot obtain the output for authentication within a particular time, the REPR packet is not transmitted. Second, the cluster head of the nearest cluster receives the REPR packet, which must validate the authentication of the REPR packet by the MAC, because the key-sharing of the inter-cluster is performed only between the cluster heads. When authentication is accomplished, the cluster head of the nearest cluster transmits the REPR packet to the earlier node. Otherwise, it must be withdrawn. Among the two cluster heads of the nearest cluster nodes, the transmission of the REPR packet is accepted instantly when the REQR packet is received, although the packet does not possess the path without a black-hole attack. Here, to detect this attack, the random mechanism for the authentication of data is used. With this mechanism, a source node is chosen at random. The message to the receiving node is controlled by the source node and transmits the authentication. The authentication comprises the MAC that uses the key-sharing between the transmitter and the receiver node. When the transmitter receives authentication along with the validation, it is then considered to be successful against a black-hole attack. The path is considered to possess the node of the black hole within it. REPR of the prohibited node is not admitted with the source node. An appropriate routing table is built during the discovery of a secure route. The table contains the route to the sink node along with information about the next hop.

### 3.4. Protocol Description

**Request for the Route**: Generate the identification of the route for transmitting REQR by Transmitter node (T):

  T → *:IDT|IDsink|IDREQR|Seq|NT|

MAC(KT, IDT|IDsink|IDREQR|Seq|NT) random number is defined by IDREQR, where Seq is the sequence number. The cluster head (node C) receives it, and six cases will be considered:

**Circumstance 1**: The cluster head will withdraw when receiving REQR, while the parent of the node is not transmitting.

**Circumstance 2**: The cluster head will withdraw when receiving REQR, while the parent of the node is transmitting and is a known packet. If the packet has not been received previously, the routing table will be updated with IDsend, IDS, IDsink, and IDREQR. Here, IDsend denotes the ID of the sender node. REQR is then sent as follows:

I → *:IDI|IDS|IDsink|IDREQR|Seq|NS|MAC(KS, IDS|IDsink|IDREQR|Seq|NS)

**Circumstance 3**: When the cluster head generates a REQR initially, and if it is not recognized as a child for the transmitter node, or if it has accepted the REQR before, then it withdraws it.

**Circumstance 4**: When the cluster head generates a REQR initially, and if it is represented as a child for the transmitter node, or if it has accepted the REQR before, then their routing table is updated with IDsend, IDS, IDsink, and IDREQR. In addition, it forwards the REQR:

I → *:IDI|IDG|IDS|IDsink|IDREQR|Seq|NS|

MAC(KS, IDS|IDsink|IDREQR|Seq|NS)

**Circumstance 5**: When the cluster head receives REQR and has been accepted earlier, then it is withdrawn.

**Circumstance 6**: When the cluster head receives REQR, which is not accepted earlier, then the routing table is checked when defining new possible routes. If there is a new route, then REPR is generated for the transmitter node T. If there is no new route, then the routing table is updated with IDsend, IDS, IDsink, and IDREQR. The updated REQR is sent as follows:

G → *:IDG|IDs|IDsink|IDREQR|Seq|NS| MAC(KS, IDS|IDsink|IDREQR|Seq|NS)

Once the receiver node accepts the REQR, the secret key KS fK (IDS0) 0 for the transmitter node has been derived. When a similar route is greater than the Seq of their routing table, then the routing table is updated and the IDS0, IDREQR, Seq, as well as the ID of the earlier cluster head, is collected. Finally, the transmission node transmits REPR.

**Reply for the Route**: When REPR is initiated by the receiver node, it transmits:

Receiver → C: IDE|IDC|IDR|ID receiver |IDREQR|Seq|N|MAC(K<C, receiver>, IDR|ID receiver|IDREQR| Seq|N|IDC)|MAC(KR, IDR| ID receiver|IDREQR|Seq|N)

The earlier node ID is given as IDE, the earlier cluster head ID is given by IDC and K < C, then the RERP packet is created by the key-sharing of the inter-cluster then,

C → B: IDE|IDB|IDC|IDC2|IDR|ID receiver|IDREQR|Seq|N|MAC(KBC, IDR|ID receiver|DREQR|Seq|N|IDC2|IDC|IDB)|

The earlier node ID is given as IDE, earlier cluster head B ID is given by IDB, transmitting cluster head C ID is given by IDC then the next node’s ID is C2 ID.

The cluster head of the subsequent hop transmits the authentication output to an earlier node of the cluster head and is given as:

C2 → C1: C1 ID | C2 ID|IDR|ID receiver|Seq|Rverify|MAC(KC1C2, IDR|ID receiver|Seq|IDC1|IDC2|Rverify)

The verification result is given by Rverify. When C2 has renewed a possible route to the receiver, then Rverify says YES. Else, it will say NO. When the result of the verification is NO, then the subsequent hop of the cluster head does not have a possible route, and REPR is withdrawn by C1. When the result of the verification is YES, then there is a new route for the cluster head. REPR is transmitted by the earlier node. All these operations will be updated in the routing table by the central node. If REPR is accepted by the cluster head, then the initial MAC is authenticated with the subsequent cluster head. When authentication is not passed, then it withdraws. When the ID of the earlier cluster head integrated by REPR is not the present cluster head’s ID, then it withdraws. Otherwise, the routing table is updated. At that point, when the REPR is initially from the receiver node, the cluster head transmits the REPR as

B → A: IDB1 |IDA|IDB| IDB2|IDR| ID receiver |IDREQR|Seq|N|MAC(KAB|IDR|ID receiver|IDREQR|Seq|N|B2 ID|IDB|IDA)|MAC(KS, IDR|ID receiver|IDREQR|Seq|N)

where B1 ID and B2 ID denote the ID of the previous node B1 and next node B2, the adjacent group leaders are indicated as A and B. If REPR originates from the intermediate group leader C, then REPR is sent as follows:

B → A: B1 ID|IDA|IDB|B2 ID|IDC|IDS|IDsink|IDREQR|Seq|N|

MAC(KAB, IDC|IDS|IDsink|IDREQR|Seq|N|B2 ID|IDB|IDA)

where B1 ID and B2 ID denote the ID of the previous node B1 and next node B2, the adjacent group leaders are indicated as A and B and IDC denotes the ID of the node that generates REPR.

Acknowledgment of Data: The authentication from the receiver node is transmitted as follows: Receiver → S: IDP| IDR| ID receiver |NR|NReceiver|

MAC(KS, IDR| ID receiver |NR|NReceiver)

**Figure 6:**
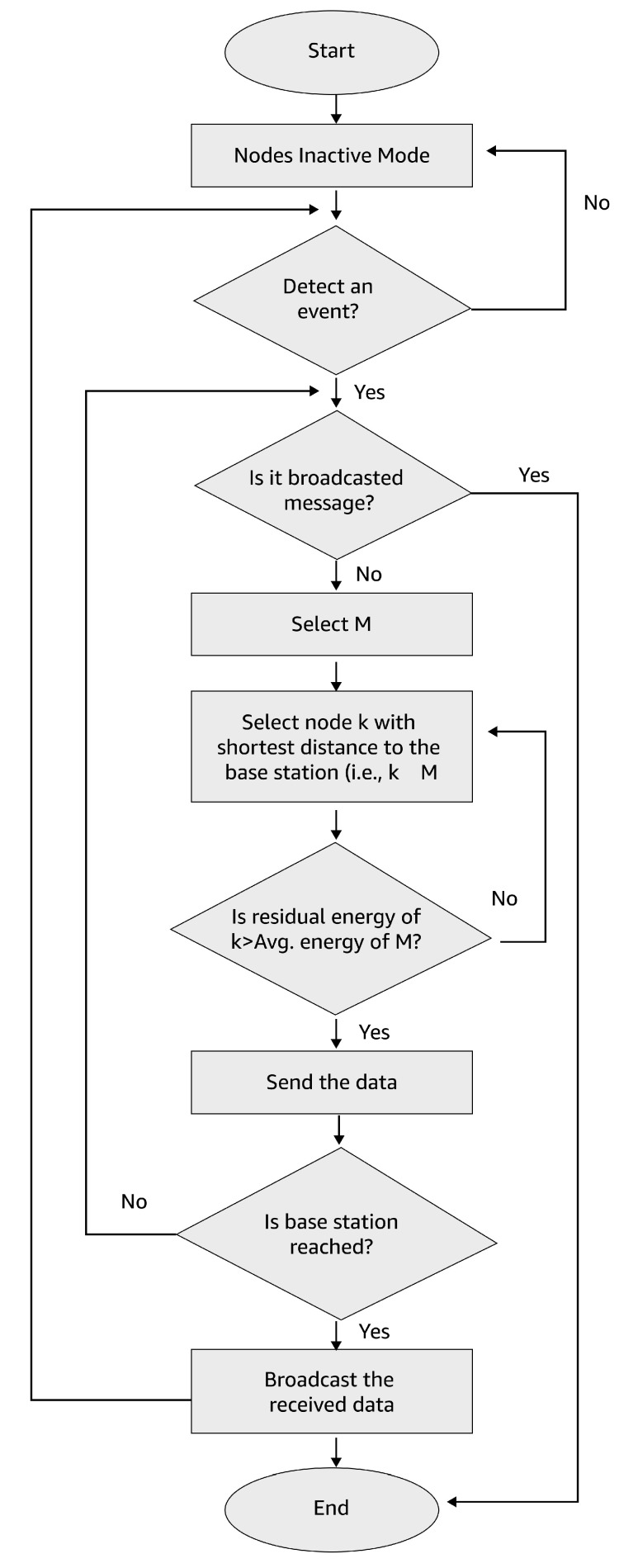
Flowchart for the routing protocol.

The ID of the preceding hop is given by IDP in the route. When the authentication is accepted by the transmitter node, and validation is passed by the MAC, then that route is safer from a black-hole attack. Simply, the receiver node generates the appropriate MAC through the secret key KS. When the validation fails, the transmitter node would be suspicious, regardless of whether the route is secured against a black-hole attack or not. For further confirmation, the transmitter node transmits additional control messages often at the rate of the message as Rc’ (Rc < Rc’ < Rd). When the appropriate authentication is not received within the threshold time, the transmission node confirms that the route is not safe. The cluster head that creates REPR must then be added to the black-hole list. The route is protected from black-hole attacks when data authentication is accepted within the threshold time. The SOPR algorithm is described in Algorithm 1, and the routing process is described in Figure 6.
**Algorithm 1** Algorithm for SOPR.
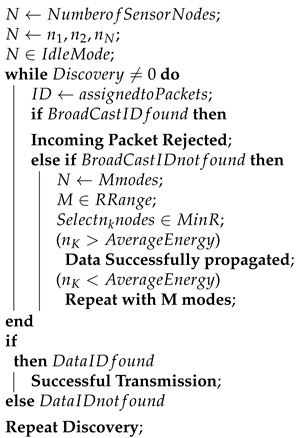


### 3.5. Energy Efficiency Using Balanced Energy-Efficient and Reliable Algorithm

In this model, the energy is dissipated from the transmitter and receiver for the operation of power amplifiers and electronic circuits. Equations (1) and (2) indicate energy consumption when sending and receiving n-bit data over a distance d0 that exists between the transmitter and receiver. According to the energy model, each node’s energy consumption should be proportional to 2d if the distance between the transmitter and receiver is small compared to the threshold distance d0; otherwise, energy consumption is proportional to 4d.

Energy Model: The energy model in WBAN is designed at the physical and MAC layers for the following reasons: In the physical layer, there is a loss due to channel distance, and in the MAC layer, the energy efficiency is affected due to idle listening, overhearing, etc. These factors are considered when estimating the energy loss of every sensor node during communication. The two-channel propagation models used are the free-space and the multipath fading channel model. The former is used for direct transmission power loss (one hop) and the latter is used for packet transmission via multi-hop power loss. As a result, the amount of energy required to send an l-bit packet over distance d is calculated as per Equation (1).

(1) $$E_{TX}\left(l,d\right)\;=\;\left(lE_{elec}+l_{fs}d^{2},d\;<\;0\right)\left(lE_{elec}+l_{mp}d^{4},d\;>\;0\right)$$

where free-space energy loss is denoted by efs, multipath energy loss is denoted by emp, the distance between the source and the destination node is denoted by *d*, and crossover distance is denoted by Equation (2)

(2) $$d_{0}=\sqrt{e_{fs}/e_{mp}}$$

The energy required by the radio to receive the message is calculated as per Equation (3).

(3) $$E_{R}X\left(l\right)=lE_{elec}$$

Therefore, the physical and MAC layers of WBAN are designed with respect to the transmitting and receiving of power energy levels. The energy model in this proposed methodology uses the Balanced Energy-Efficient and Reliable (BEER) algorithm, which is used for transmission within the cluster heads of a network. There will be an improvement in data transmission when the network’s cluster heads’ routing is optimized, and this includes a Balanced Energy-Efficient and Reliable (BEER) algorithm that will optimize energy consumption. This will increase network throughput and energy efficiency while decreasing end-to-end delay. Routing with SOPR will minimize the traffic within the network and enhance the packet-delivery ratio.

## 4. Results

A Network Simulator (ns-2.34) is used to create the wireless network environment. A network environment that explores the potential of SOPR-BEER is planned for the development of a Secure Optimal Path-Routing Protocol (SOPR) along with a Balanced Energy-Efficient and Reliable (BEER) algorithm to save energy. The simulation is executed in the environment with 100 nodes with a 250 × 250 square-meter terrain area. The environment uses a wireless channel and omnidirectional antenna. The results are compared with the existing works of [44,45]. The simulation parameters for the network and the SOPR-BEER parameters are highlighted in Table 2.

**Figure 7:**
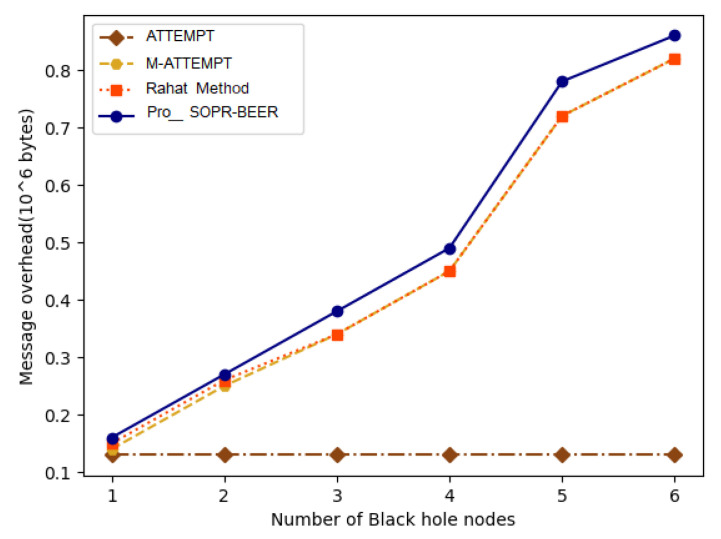
Route-discovery overheads.

The existing works discussed the increasing number of black-hole triggers, in addition to route detection. In Figure 7, pro-SOPR-BEER, the average time to find a black hole indicates that adversary nodes masquerading as black holes are detected faster than ATTEMPT, M-ATTEMPT, and algorithm proposed in [45] The detection is faster because black-hole discovery is only performed locally in pro-SOPR-BEER. However, alternative path requests are sent from the source node to the intermediate node’s next hop in ATTEMPT, M-ATTEMPT, and algorithm proposed in [45] to check the route authenticity. The discovery of the black-hole node is significantly delayed by this method of verification.

In Figure 7, the overhead message to create a secure path against numerous non-cooperative black-hole attacks is displayed. Although data are being transmitted, there exist packet overheads over the communication network. The overhead in the existing work involves the message overhead in the response to the route and in the exploration of the path. Even when the number of attack nodes surges, the message overhead for pro-SOPR-BEER is almost the same, which remains consistent with the result of a computational cost analysis of T(N), where N denotes the number of sensor nodes in the wireless environment. However, it is significantly less than in existing systems. The above process is due to the black-hole node being found locally in pro-SOPR-BEER. However, in the existing work, if the route response is received by the source node, a route request is additionally sent to the black-hole node’s subsequent hop to validate the route response. Consistent with the results of computational cost analysis in existing studies, increasing the number of attacks results in the detection of additional routes.

**Figure 8:**
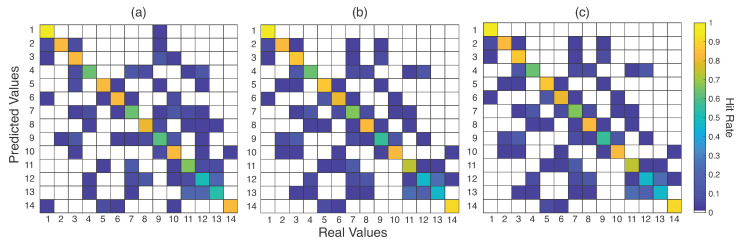
Comparison of mean time to detect the black holes.

In Figure 8 pro-SOPR-BEER, the average time to find a black-hole node indicates that adversary nodes masquerading as black holes are detected faster compared to existing works. The detection of the black hole is faster because the discovery is performed locally in pro-SOPR-BEER. However, the source sensor node initiates an alternative path request to the intermediate node’s next hop in ATTEMPT and M-ATTEMPT to check the route authenticity. The discovery of the black-hole node is significantly delayed by this method of verification.

## 5. Discussion

### 5.1. Performance Comparison of Sensor Nodes with Black-Hole Nodes

**Figure 9:**
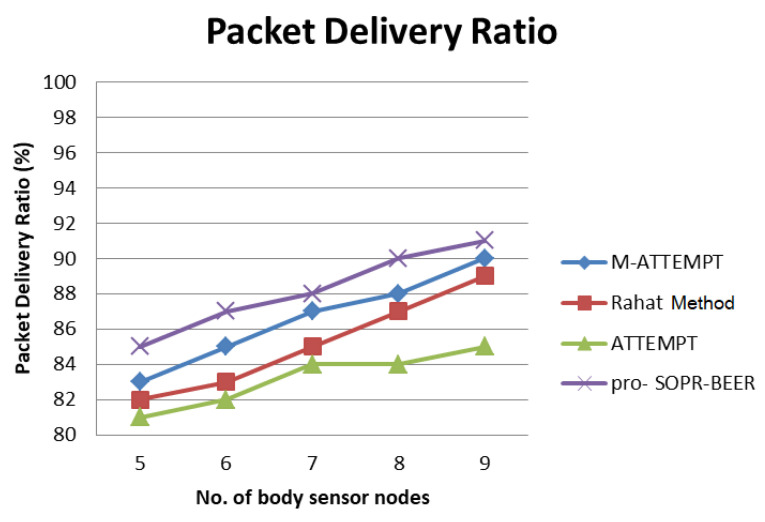
Packet-delivery ratio.

Figure 9 shows the packet-delivery ratio metric-based comparison of the existing body-area sensor network routing algorithms, namely M-ATTEMPT and ATTEMPT, and the proposed secure routing algorithm, namely pro-SOPR-BEER. This comparison has been considered for 5, 6, 7, 8, and 9 sensor nodes that were fixed on the body to perform the routing of collected data.

It is inferred from Figure 9 that the packet-delivery ratio provided by the proposed pro-SOPR-BEERis higher for all the experiments conducted with a varying number of sensors in WBAN, compared with the existing protocols, namely M-ATTEMPT and ATTEMPT. This performance improvement was achieved in the proposed model, namely pro_SOPR_BEER, for the application of security using encryption and shared secret keys. The encrypted data could not be analyzed by the sensor nodes. In addition, existing data were not dropped from their malicious nodes due to their inability in finding the original contents of the data. Therefore, packet drops were reduced, leading to an increase in the packet-delivery ratio in the proposed secure routing algorithm.

The energy equation is represented in Equation (4)

(4) $$\sum E_{consumed}\;=\;\sum E_{initial}-\sum E_{remaining}$$

**Figure 10:**
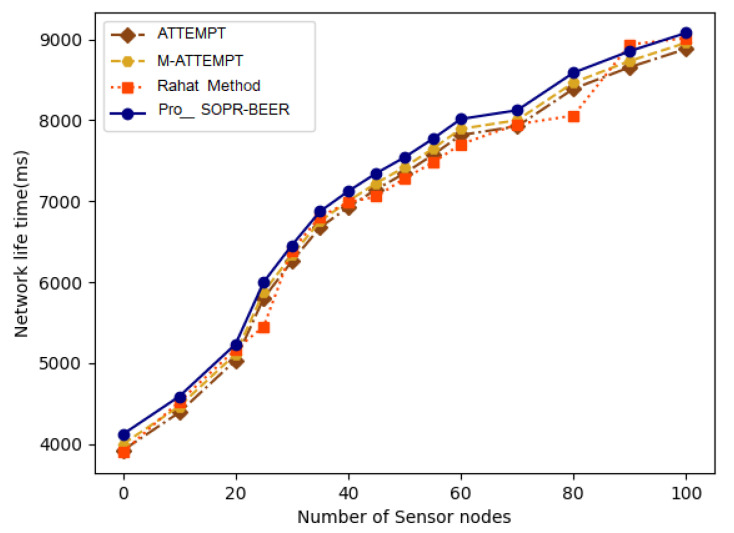
Network lifetime.

Figure 10 plots the network lifetime of different nodes. However, the graphical representation shows that the estimated network lifetime of the projected pro-SOPR-BEER is very high compared to the ATTEMPT and M-ATTEMPT processes. Additionally, this sustainable function of the proposed pro-SOPR-BEER for a safe route-discovery scheme improves the possibility of maintaining the live network nodes to boost the lifespan of the network. Compared with benchmarks ATTEMPT and M-ATTEMPT, the increase in the number of active network nodes as the basis for choosing a secure route-discovery scheme systematically increases the life of the network. The proposed pro-SOPR-BEER, therefore, retains several sensor nodes under unequal energy at an average rate of 3%, 5%, and 6% when compared to the ATTEMPT and M-ATTEMPT techniques.

### 5.2. Energy Consumption

**Figure 11:**
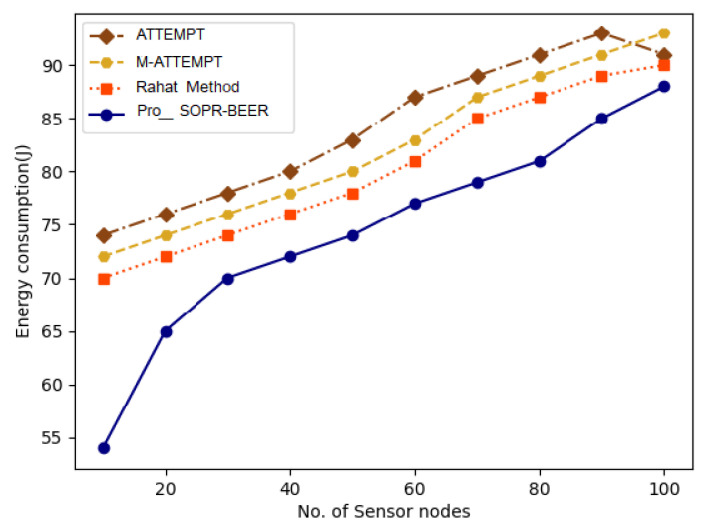
Energy consumption vs. the number of sensor nodes.

The total energy consumed for all the nodes in the network can be calculated as per Figure 11. To ensure that the proposed 100-node algorithms are also energy-efficient, their power consumption is displayed in Figure 11. Energy consumption is determined by the average energy consumption of a single sensor node. As opposed to other approaches, the proposed pro-SOPR-BEER techniques are highly energy-efficient. For instance, longer distances between the cluster head and the nodes are the main reasons for energy consumption, and, depending on the energy level of the nodes, they randomly advertise themselves as cluster heads in the proposed pro-SOPR-BEER algorithm. This minimizes the energy needed for contact within the clusters. The proposed algorithm, pro-SOPR-BEER, also minimizes the energy spent on inter-cluster communication. The performance of M-ATTEMPT is minimal when compared to the other existing schemes. Collectively, all the schemes failed to achieve better results when compared with the proposed pro-SOPR-BEER algorithm. The proposed pro-SOPR-BEER algorithm increases successful packet transmission by reducing packet loss, and the consumption of energy is greatly reduced. The energy consumption of the proposed pro-SOPR-BEER scheme is estimated for the determination of network quality.

### 5.3. Challenges

- The limited battery capacity of devices on the network can reduce the time duration that a device can remain connected to the network. As a result, this might reduce the efficiency of the routing protocol.
- Changes in the network environment can affect the efficiency of the routing protocol, such as changes in the number of nodes in the network or the network topology.
- If a link in the network fails, the routing protocol may fail to identify the correct path on which data packets to travel, resulting in decreased energy efficiency. Moreover, link failure can cause congestion and delays in the network, affecting energy efficiency.

### 5.4. Future Directions

The wireless capability of sensors to detect and transmit signals across a wide range of frequencies, and ensure that the data are transferred successfully, has been tested with a simulator. Deploying and testing the real-time environment is planned as future work. Moreover, we have planned to use fuzzy rules for trust modeling for security and effective clustering in WBAN.

## 6. Conclusions

In this paper, a Secure Optimal Path-Routing (SOPR) protocol is proposed for the identification and discovery of the routes in WBAN that are secure against black-hole attacks. In this case, the proposed protocol adopts cryptography techniques for the symmetric key when finding a secured path. Additionally, the protocol is integrated with the Balanced Energy-Efficient and Reliable (BEER) algorithm to provide reliable and safe data transmission. Most black-hole attacks are only exposed locally, except for cluster heads, who work in conjunction with other nodes to perform black-hole attacks. Therefore, the attacks are detected much more quickly and with minimal message overheads. The simulation results show that the proposed protocol, pro-SOPR-BEER, has improved performance in protecting against black-hole attacks while discovering secure routes, when compared to the ATTEMPT, M-ATTEMPT, and algorithm proposed in [45] techniques.

## Figures and Tables

**Table 1:** Table describing the parameters of the OTP algorithm.

Algorithm Type	Encryption
Input	Plain text from source file
Output	Cipher text
Assumptions	Block size = 64, key size = 512 bits, OTP—One-Time Pad, CBC—Cipher Block Chaining

**Table 2:** Simulation parameters.

Parameters Used	Value
Region of sensor fields	250 × 250
Location of the Base Station	50 × 100
Maximum number of nodes	100
Maximum number of rounds	1500
Propagation model	Free-space and Multipath fading channel model

## Data Availability

Not applicable.

## References

[1] Behera T.M., Samal U.C., Mohapatra S.K., Khan M.S., Appasani B., Bizon N., Thounthong P. (2022). Energy-Efficient Routing Protocols for Wireless Sensor Networks: Architectures, Strategies, and Performance. Electronics.

[2] Gopalakrishnan M., Arumugam G., Lakshmi K., Vel S. (2016). SAC-TA: A Secure Area Based Clustering for Data Aggregation Using Traffic Analysis in WSN. Circuits Syst..

[3] Arafat M.Y., Pan S., Bak E. (2023). Distributed Energy-Efficient Clustering and Routing for Wearable IoT Enabled Wireless Body Area Networks. IEEE Access.

[4] Azad P., Sharma V. (2013). Cluster head selection in wireless sensor networks under fuzzy environment. Int. Sch. Res. Not..

[5] Chen C., Wang L.C., Yu C.M. (2022). D2CRP: A Novel Distributed 2-Hop Cluster Routing Protocol for Wireless Sensor Networks. IEEE Internet Things J..

[6] Roshini A., Kiran K. (2023). Hierarchical energy efficient secure routing protocol for optimal route selection in wireless body area networks. Int. J. Intell. Netw..

[7] Firdous S., Bibi N., Wahid M., Alhazmi S. (2022). Efficient Clustering Based Routing for Energy Management in Wireless Sensor Network-Assisted Internet of Things. Electronics.

[8] Zaman K., Sun Z., Hussain A., Hussain T., Ali F., Shah S.M., Rahman H.U. (2023). EEDLABA: Energy-Efficient Distance-and Link-Aware Body Area Routing Protocol Based on Clustering Mechanism for Wireless Body Sensor Network. Appl. Sci..

[9] Sangeetha G., Vijayalakshmi M., Ganapathy S., Kannan A. (2020). An improved congestion-aware routing mechanism in sensor networks using fuzzy rule sets. Peer-to-Peer Netw. Appl..

[10] Logambigai R., Kannan A. (2016). Fuzzy logic based unequal clustering for wireless sensor networks. Wirel. Netw..

[11] Logambigai R., Ganapathy S., Kannan A. (2018). Energy–efficient grid–based routing algorithm using intelligent fuzzy rules for wireless sensor networks. Comput. Electr. Eng..

[12] Pandiyaraju V., Logambigai R., Ganapathy S., Kannan A. (2020). An energy efficient routing algorithm for WSNs using intelligent fuzzy rules in precision agriculture. Wirel. Pers. Commun..

[13] Gayathri A., Ruby D., Manikandan N., Gopalakrishnan T., Anusha K., Narayanasamy P. (2022). Data location integration with stable routing: Stable and optimal data transmission in wireless sensor networks. Trans. Emerg. Telecommun. Technol..

[14] Shimly S.M., Smith D.B., Movassaghi S. (2019). Experimental analysis of cross-layer optimization for distributed wireless body-to-body networks. IEEE Sens. J..

[15] Förster A., Murphy A.L., Schiller J., Terfloth K. An efficient implementation of reinforcement learning based routing on real WSN hardware. Proceedings of the 2008 IEEE International Conference on Wireless and Mobile Computing, Networking and Communications.

[16] Hu T., Fei Y. (2010). QELAR: A machine-learning-based adaptive routing protocol for energy-efficient and lifetime-extended underwater sensor networks. IEEE Trans. Mob. Comput..

[17] Patel A., Shah H.B. (2015). Reinforcement learning framework for energy efficient wireless sensor networks. Int. Res. J. Eng. Technol. IRJET.

[18] Khan F., Memon S., Jokhio S.H. Support vector machine based energy aware routing in wireless sensor networks. Proceedings of the 2016 2nd International Conference on Robotics and Artificial Intelligence (ICRAI).

[19] Kumar K.A., Avinash J., Poornima G. QoS Aware Load Balancing in Cognitive Wireless Sensor Networks using Machine Learning Concepts. Proceedings of the 2018 3rd IEEE International Conference on Recent Trends in Electronics, Information & Communication Technology (RTEICT).

[20] Singh K., Kaur J. (2017). Machine learning based link cost estimation for routing optimization in wireless sensor networks. Adv. Wirel. Mob. Commun..

[21] Masoud M.Z., Jaradat Y., Jannoud I., Al Sibahee M.A. (2019). A hybrid clustering routing protocol based on machine learning and graph theory for energy conservation and hole detection in wireless sensor network. Int. J. Distrib. Sens. Netw..

[22] Murudkar C.V., Gitlin R.D. Optimal-capacity, shortest path routing in self-organizing 5G networks using machine learning. Proceedings of the 2019 IEEE 20th Wireless and Microwave Technology Conference (WAMICON).

[23] Hendriks T., Camelo M., Latré S. Q 2-routing: A Qos-aware Q-routing algorithm for wireless ad hoc networks. Proceedings of the 2018 14th International Conference on Wireless and Mobile Computing, Networking and Communications (WiMob).

[24] Vimalapriya M., VigneshBaalaji S., Sandhya S. (2019). Energy-Centric Route Planning using Machine Learning Algorithm for Data Intensive Secure Multi-Sink Sensor Networks. Int. J. Innov. Technol. Explor. Eng. (IJITEE).

[25] Yang J., He S., Xu Y., Chen L., Ren J. (2019). A trusted routing scheme using blockchain and reinforcement learning for wireless sensor networks. Sensors.

[26] Yao H., Yuan X., Zhang P., Wang J., Jiang C., Guizani M. A machine learning approach of load balance routing to support next-generation wireless networks. Proceedings of the 2019 15th International Wireless Communications & Mobile Computing Conference (IWCMC).

[27] Ghaffari A. (2017). Real-time routing algorithm for mobile ad hoc networks using reinforcement learning and heuristic algorithms. Wirel. Netw..

[28] Strykhaliuk B., Kolodiy R., Faichuk V. Method for Intelligent Routing Within Ad-Hoc Networks with Complex Topology. Proceedings of the 2019 3rd International Conference on Advanced Information and Communications Technologies (AICT).

[29] FatemiAghda S.A., Mirfakhraei M. (2019). An improved cluster routing protocol to increase the lifetime of wireless sensor network (WSN). Wirel. Pers. Commun..

[30] Vinodhini R., Gomathy C. (2020). MOMHR: A dynamic multi-hop routing protocol for WSN using heuristic based multi-objective function. Wirel. Pers. Commun..

[31] Rodrigues P., John J. (2020). Joint trust: An approach for trust-aware routing in WSN. Wirel. Netw..

[32] Panchal A., Singh L., Singh R.K. RCH-LEACH: Residual energy based cluster head selection in LEACH for wireless sensor networks. Proceedings of the 2020 International Conference on Electrical and Electronics Engineering (ICE3).

[33] Sixu L., Muqing W., Min Z. FMUCR: Fuzzy-based multi-hop unequal cluster routing for WSN. Proceedings of the 2020 IEEE Wireless Communications and Networking Conference (WCNC).

[34] Naushad A., Abbas G., Shah S.A., Abbas Z. Energy efficient clustering with reliable and load-balanced multipath routing for WSNs. Proceedings of the 2020 3rd International Conference on Advancements in Computational Sciences (ICACS).

[35] Aalsalem M.Y., Khan W.Z., Saad N., Hossain M., Atiquzzaman M., Khan M.K. (2016). A new random walk for replica detection in WSNs. PLoS ONE.

[36] Osanaiye O., Alfa A.S., Hancke G.P. (2018). A statistical approach to detect jamming attacks in wireless sensor networks. Sensors.

[37] Mahdi O.A., Al-Mayouf Y.B., Ghazi A.B., Wahab A.A., Idris M. (2018). An energy-aware and load-balancing routing scheme for wireless sensor networks. Indones. J. Electr. Eng. Comput. Sci..

[38] Abdulkareem K.H., Mohammed M.A., Gunasekaran S., Al-Mhiqani M.N., Mutlag A.A., Mostafa S.A., Ali N., Ibrahim D.A. (2019). A review of fog computing and machine learning: Concepts, applications, challenges, and open issues. IEEE Access.

[39] Khadidos A.O., Shitharth S., Khadidos A.O., Sangeetha K., Alyoubi K.H. (2022). Healthcare data security using IoT sensors based on random hashing mechanism. J. Sens..

[40] Shitharth S., Kshirsagar P.R., Balachandran P.K., Alyoubi K.H., Khadidos A.O. (2022). An innovative perceptual pigeon galvanized optimization (PPGO) based likelihood Naïve Bayes (LNB) classification approach for network intrusion detection system. IEEE Access.

[41] Xiong H., Jin C., Alazab M., Yeh K.H., Wang H., Gadekallu T.R., Wang W., Su C. (2021). On the design of blockchain-based ECDSA with fault-tolerant batch verification protocol for blockchain-enabled IoMT. IEEE J. Biomed. Health Inform..

[42] Wang W., Chen Q., Yin Z., Srivastava G., Gadekallu T.R., Alsolami F., Su C. (2021). Blockchain and PUF-based lightweight authentication protocol for wireless medical sensor networks. IEEE Internet Things J..

[43] Khadidos A.O., Manoharan H., Selvarajan S., Khadidos A.O., Alyoubi K.H., Yafoz A. (2022). A classy multifacet clustering and fused optimization based classification methodologies for SCADA security. Energies.

[44] Javaid N., Abbas Z., Fareed M., Khan Z.A., Alrajeh N. (2013). M-ATTEMPT: A new energy-efficient routing protocol for wireless body area sensor networks. Procedia Comput. Sci..

[45] Khan R.A., Mohammadani K.H., Soomro A.A., Hussain J., Khan S., Arain T.H., Zafar H. (2018). An energy efficient routing protocol for wireless body area sensor networks. Wirel. Pers. Commun..

